# Supplementary material for: ProteinShader: illustrative rendering of macromolecules
Source: BMC Struct Biol. 2009 Mar 30;9:19. doi: 10.1186/1472-6807-9-19 (PMC2672931; doi:10.1186/1472-6807-9-19)
Supplement: Additional file 1 — ProteinShader program without source code. This compressed file contains the complete ProteinShader program including associated libraries, but no source code. A README.txt file gives an overview of the ProteinShader distribution, and the index.html file in the help subdirectory has directions on getting started with the program as well as a set of tutorials. [file 1472-6807-9-19-S1.zip › ProteinShader-beta-0_9_4-binary/help/api/org/proteinshader/gui/components/menubar/package-use.html]

Uses of Package org.proteinshader.gui.components.menubar (ProteinShader API)


|  |  |  |
| --- | --- | --- |
| **Overview** \| **Package** \| Class \| **Use** \| **Tree** \| **Deprecated** \| **Index** \| **Help** | |  |
| PREV   NEXT | **FRAMES**    **NO FRAMES**     **All Classes** |  |


---


## **Uses of Package org.proteinshader.gui.components.menubar**

| Packages that use org.proteinshader.gui.components.menubar | |
| --- | --- |
| **org.proteinshader.gui** | Holds all of the Swing GUI components and their associated listeners, including class Renderer, which is registered as a listener for the GLCanvas object that is used a drawing surface. |
| **org.proteinshader.gui.components.menubar** | Holds the JMenus used in menu bar above the canvas. |
| **org.proteinshader.gui.listeners.menubar** | Holds the listeners for the items used in the JMenus of the menu bar above the canvas. |

Classes in org.proteinshader.gui.components.menubar used by org.proteinshader.gui

****MainMenuBar****
  
          Provides the menu bar to place at the top of the ProteinShader
programs's main GUI window.

Classes in org.proteinshader.gui.components.menubar used by org.proteinshader.gui.components.menubar

****HelpMenu****
  
          Provides an "About" box and help for using the ProteinShader program.

****StyleMenu****
  
          Provides a menu for selecting the style to use when displaying a structure:
Cartoon (Tubes or Ribbons) or Atom (Space Filling, Balls-andSticks, or Sticks).

****ToolsMenu****
  
          Provides the Tools menu for the MainMenuBar, which can be used to open
and close the control panel on the right side of the GUI.

****VisibilityMenu****
  
          Provides the Vi menu for the MainMenuBar.

Classes in org.proteinshader.gui.components.menubar used by org.proteinshader.gui.listeners.menubar

****AboutBox****
  
          A simple dialog box that displays a title, an image (PNG, GIG, or JPEG), and a
close button.

****BackgroundMenu****
  
          Provides the 'Background' menu of the MainMenuBar, which allows the
user to change the background color of the canvas.

****HelpMenu****
  
          Provides an "About" box and help for using the ProteinShader program.

---


|  |  |  |
| --- | --- | --- |
| **Overview** \| **Package** \| Class \| **Use** \| **Tree** \| **Deprecated** \| **Index** \| **Help** | |  |
| PREV   NEXT | **FRAMES**    **NO FRAMES**     **All Classes** |  |


---

# *Copyright © 2007-2008*
